# STAT1 Mediates Oroxylin A Inhibition of iNOS and Pro-Inflammatory Cytokines Expression in Microglial BV-2 Cells

**DOI:** 10.1371/journal.pone.0050363

**Published:** 2012-12-06

**Authors:** Po-Wen Liu, Mei-Fang Chen, Andy Po-Yi Tsai, Tony J. F. Lee

**Affiliations:** 1 Institute of Pharmacology and Toxicology, Tzu Chi University, Hualien, Taiwan; 2 Department of Life Sciences, Tzu Chi University, Hualien, Taiwan; 3 Center for Vascular Medicine, College of Life Sciences, Tzu Chi University, Hualien, Taiwan; 4 Department of Research, Buddhist Tzu Chi General Hospital, Hualien, Taiwan; 5 Tzu Chi College of Technology, Hualien, Taiwan; 6 Department of Pharmacology, Southern Illinois University School of Medicine, Springfield, Illinois, United States of America; Institute Biomedical Research August Pi Sunyer (IDIBAPS) - Hospital Clinic of Barcelona, Spain

## Abstract

Microglia-mediated inflammation is implicated in pathogenesis of neurodegenerative diseases. Oroxylin A, a flavonoid isolated from *Scutellariae baicalensis*, has been shown to ameliorate microglia activation-mediated neurodegeneration *in vivo*. The molecular mechanism underlying the inhibitory effects of oroxylin A on microglia activation, however, remains unknown. In the present study, effects of oroxylin A co-treated with lipopolysaccharide (LPS, 100 ng/ml) on LPS-induced activation of cultured microglial BV-2 cells were examined. Nitric oxide (NO) production was determined by Greiss method. Expression of inducible nitric oxide synthase (iNOS), interleukin (IL)-1β and IL-6 was assessed using real-time RT-PCR or Western blot analysis. Furthermore, activation of the nuclear factor κB (NFκB) and the signal transducer and activator of transcription 1 (STAT1) was examined by Western blot analysis and transcription factor DNA-binding activity assay. Our results indicated that oroxylin A (10–100 µM) in a concentration-dependent manner inhibited LPS-induced NO production via blocking iNOS expression at both mRNA and protein levels without affecting the degradation rate of iNOS mRNA. Moreover, oroxylin A significantly attenuated LPS-induced late expression (20 hours after LPS challenge) of IL-1β and IL-6. Furthermore, oroxylin A significantly suppressed LPS-induced JAK2-mediated STAT1 phosphorylation without affecting LPS-induced NFκB-p65 nuclear translocation or NFκB-p65 DNA-binding activity. This is consistent with the finding that AG490, a specific JAK2 inhibitor, significantly inhibited LPS-induced STAT1 phosphorylation with almost completely diminished iNOS expression. These results suggest that oroxylin A, via suppressing STAT1 phosphorylation, inhibits LPS-induced expression of pro-inflammatory genes in BV-2 microglial cells.

## Introduction

Accumulating evidence suggests that inflammation play a critical role in neurodegenerative diseases, including Parkinson's disease [1], Alzheimer's disease [2], Huntington's disease [3] and multiple sclerosis [4]. The inflammation in the central nervous system (CNS) is primarily mediated by microglia [5] which are resident innate immune cells in the CNS. Microglia are readily activated by danger signals, such as molecules released from damaged cells or components found on pathogens [6]. Activation of microglia is indispensible for clearance of cell debris or invading pathogens [5], [7]. However, the prolonged and massive activation of microglia with excessive production of pro-inflammatory factors is thought, in part, responsible for inflammation-induced neurodegeneration [6], [8].

Nitric oxide (NO), produced by inducible nitric oxide synthase (iNOS) in microglia, is one of the best characterized pro-inflammatory factors that induce neuronal death. It has been demonstrated that iNOS-deficient mice exhibited less neuronal loss in a Parkinson's disease animal model [9]. Moreover, inhibition of iNOS prevented microglia-mediated neuronal death, indicating that NO plays a pivotal role in microglia-mediated neurotoxicity [10]. In addition to NO, interleukins (ILs) such as IL-1β and IL-6 also play critical roles in microglia-mediated neurodegeneration [8], [11], [12].

The signal transductions in the intracellular milieu induced by lipopolysaccharide (LPS), a bacterial endotoxin widely used for studying experimental inflammation, eventually lead to the activation of transcription factors, including nuclear factor κB (NFκB) and signal transducer and activator of transcription 1 (STAT1), which mediate the expression of iNOS and interleukins. NFκB, which exists primarily as a p50/p65 heterodimer, is retained in the cytoplasm through its association with inhibitory κB (IκB) [13]. LPS induces the degradation of IκB, which leads to the nuclear translocation of NFκB, resulting in the transcription of NFκB-responsive genes. Likely, LPS induces phosphorylation of STAT1, resulting in the dimerization and nuclear translocation of STAT1, followed by transcription of STAT1-responsive genes. STAT1 phosphorylation induced by LPS requires *de novo* synthesis of interferons (IFNs) [14], which in an autocrine/paracrine manner triggers the activation of IFN receptors, resulting in the recruitment and the activation of Janus kinase (JAK), and, in turn, stimulating the phosphorylation of STAT1 [15]. Accordingly, activation of STAT1 in response to LPS is delayed compared with that of NFκB. Although several plant flavonoids have been reported to attenuate the expression of iNOS and interleukins via inhibiting NFκB activation [16], it has also been demonstrated that extracts of green tea and American ginseng preferentially suppress the activation of STAT1, but not that of NFκB, in inhibiting iNOS expression in macrophages and epithelial cells [17], [18]. Hence, we are interested in the roles of NFκB and STAT1, and their influence by plant flavonoids in mediating expression of iNOS and/or interleukins in different cell types such as microglia.

Oroxylin A, 5,7-Dihydroxy-6-methoxyflavone, was isolated from herbal medicine *Scutellariae baicalensis* (*S. baicalensis*). Our previous study demonstrated that oroxylin A suppressed LPS-induced iNOS and cyclooxygenase-2 expression through inhibiting the activation of NFκB-p65 in RAW264.7 macrophages [19]. Also, it was recently reported that oroxylin A and its analogues exhibited strong inhibitory activities against LPS-induced NO production in microglia [20]. Furthermore, results from *in vivo* studies indicated that oroxylin A prevented cerebral hypoperfusion-induced neuronal damage [21], and that oroxylin A ameliorated amyloid (Aβ)-induced memory impairment [22]. Oroxylin A, therefore, exhibits anti-inflammatory and neuroprotective effects [16]. Whether oroxylin A works by inhibiting the expression of pro-inflammatory genes in microglia to reduce neuronal damage, however, remains unclarified.

In the present study, we aimed to examine the molecular mechanisms by which oroxylin A inhibited LPS-induced activation of microglial BV-2 cells. Our results indicated that oroxylin A, via inhibiting STAT1 phosphorylation, blocked LPS-induced expression of pro-inflammatory genes, including iNOS, IL-1β and IL-6.

## Results

### Oroxylin A inhibited LPS-induced NO production and iNOS expression in BV-2 cells

**Figure 1:**
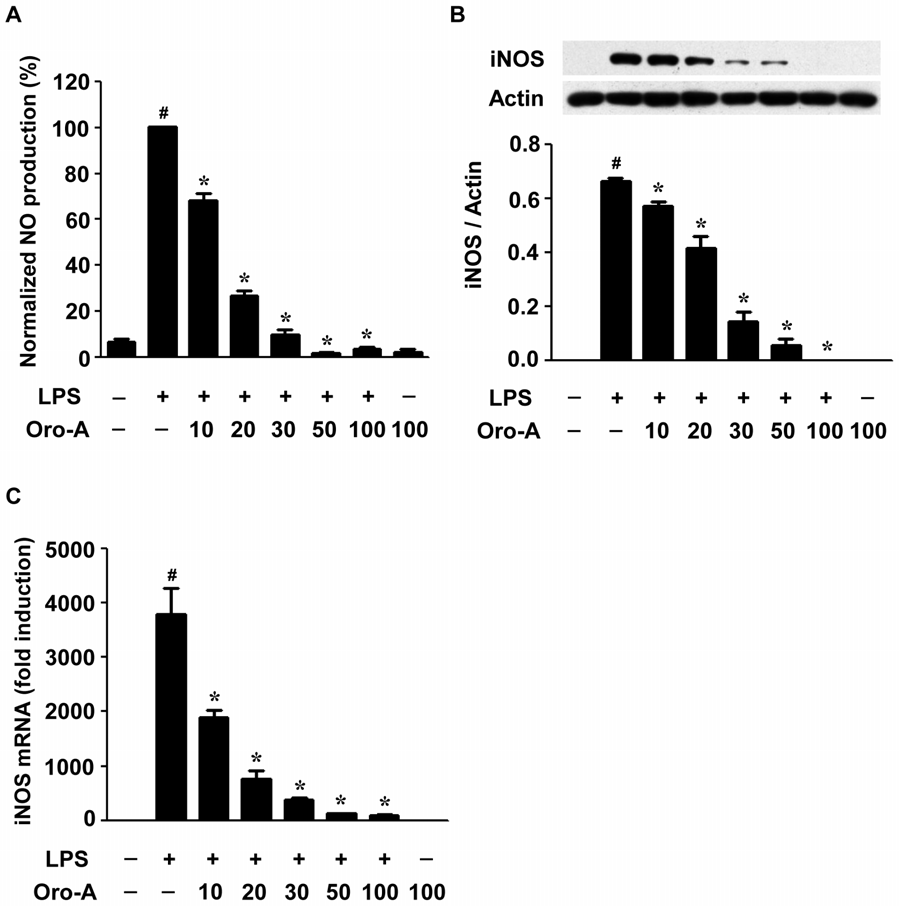
Oroxylin A attenuation of LPS-induced NO production and iNOS expression in BV-2 cells. (**A**) BV-2 cells were cultured in a 24-well plate at 5×10^4^ cells/well and were exposed to oroxylin A (Oro-A, 10–100 µM) and LPS (100 ng/ml) for 24 hours. The NO concentration in the medium was measured by Griess reaction. All results are normalized to LPS-treated control group and are means±SEM from three independent experiments in triplicates. (**B**) BV-2 cells (2×10^6^ cells) cultured in a 10-cm Petri-dish were treated with LPS (100 ng/ml) and oroxylin A (10–100 µM). After 24 hours, cells were harvested and total proteins were subjected to Western blot analysis using specific antibodies against iNOS and actin. A representative Western blot for iNOS and actin is shown in upper panel. The ratios of iNOS to actin are shown in bottom panel. Results are means±SEM from three independent experiments. (**C**) BV-2 cells (5×10^4^ cells/well) were incubated with LPS (100 ng/ml) and oroxylin A (10–100 µM) for 20 hours. Total mRNA was then isolated and converted to cDNA, followed by quantification of iNOS mRNA levels by real-time PCR. Levels of iNOS mRNA are normalized to that of actin mRNA. Results are expressed as fold induction relative to vehicle-treated cells, and are mean±SEM from three independent experiments. #, p<0.05, one-way ANOVA followed by Tukey's post hoc test compared with vehicle-treated cells. *, p<0.05, one-way ANOVA followed by Tukey's post hoc test compared with LPS-treated cells.

Oroxylin A (10–100 µM) in a concentration-dependent manner attenuated LPS (100 ng/ml)-induced NO production in BV-2 cells, with maximum inhibition at 50 µM (Fig. 1A). LPS-induced increase of iNOS proteins also was reduced by oroxylin A (10–100 µM) in a concentration dependent manner with maximum inhibition at 100 µM (Fig. 1B). In parallel, LPS-induced up-regulation of iNOS mRNA was suppressed by oroxylin A in a concentration dependent manner with maximum suppression at 50 µM (Fig. 1C).

### Oroxylin A did not affect the degradation rate of iNOS mRNA

**Figure 2:**
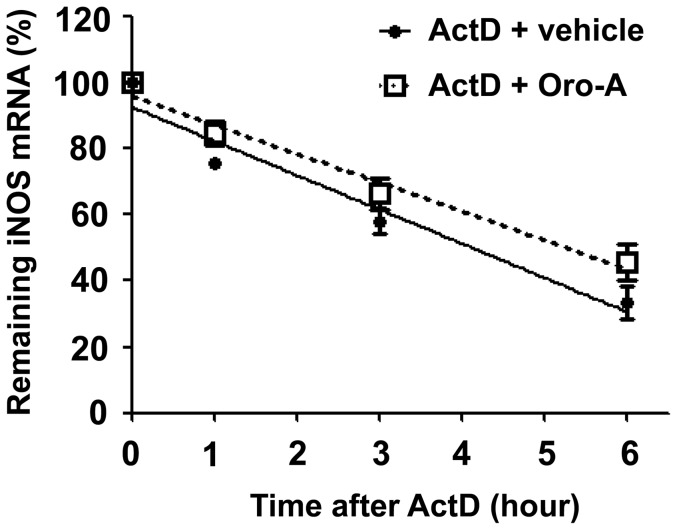
Lack of effect of oroxylin A on iNOS mRNA stability in BV-2 cells. BV-2 cells (5×10^4^ cells/well) were treated with LPS (100 ng/ml) for 20 hours. Subsequently, cells were incubated with ActD (0.1 µg/ml), a transcription inhibitor, and oroxylin A (Oro-A, 50 µM)/vehicle for indicated time periods. Total mRNA was then isolated and iNOS mRNA levels were quantified by real-time RT-PCR. The iNOS mRNA level when ActD was added was regarded as 100%, and the linear regression against time was plotted. Results are means±SEM from three independent experiments.

20 hours after LPS (100 ng/ml) stimulation, all transcriptional activity was stopped by Actinomycin D (ActD, 0.1 µg/ml). The level of iNOS mRNA at the time of ActD addition was regarded as 100%, and the decay of iNOS mRNA against time was shown in Fig. 2. In the presence of oroxylin A (50 µM), the half-life of iNOS mRNA was not significantly different from that of vehicle groups (t_1/2_ value of 5.3±0.5 for oroxylin A vs. t_1/2_ value of 4.1±0.3 for vehicle, p>0.05).

### Oroxylin A did not affect BV-2 cell viability

**Figure 3:**
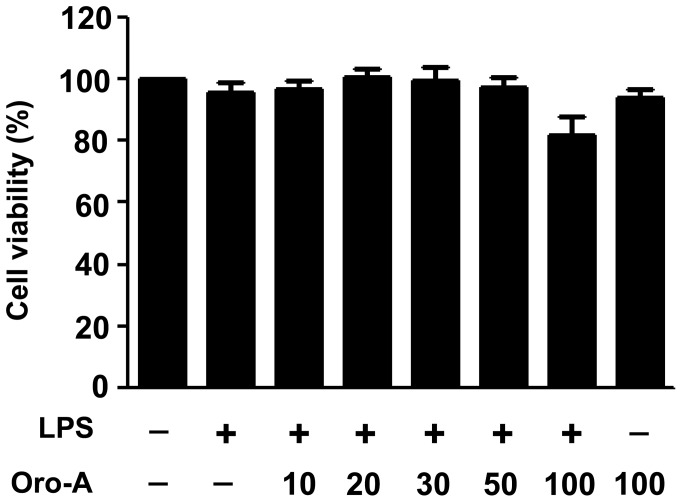
Effects of oroxylin A on cell viability. BV-2 cells (5×10^4^ cells/well) were exposed to LPS (100 ng/ml) and oroxylin A (Oro-A, 10–100 µM) for 24 hours. Subsequently, the cell viability was determined by MTT assay. Results are expressed as percent of vehicle-treated group and are means±SEM from three independent experiments in triplicates.

Oroxylin A with concentrations up to 100 µM did not significantly affect the viability of BV-2 cells in the presence of LPS (100 ng/ml) compared to the LPS-treated control group (Fig. 3). Oroxylin A at 100 µM alone did not affect the cell viability compared to the vehicle-treated group.

### Oroxylin A inhibited the late expression of IL-1β and IL-6 in BV-2 cells

**Figure 4:**
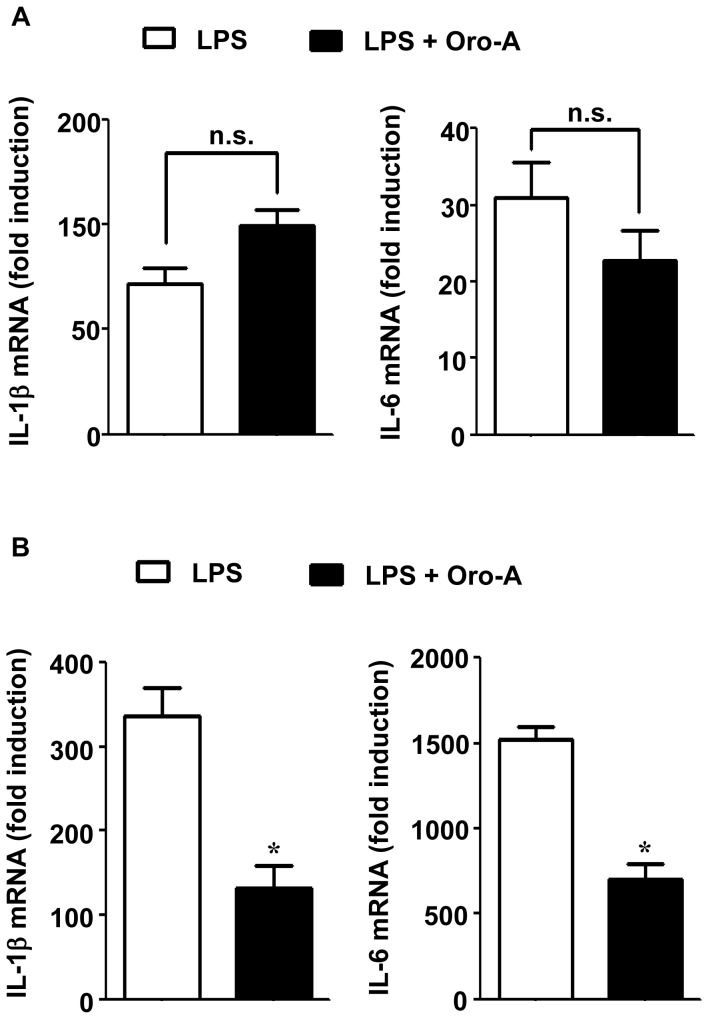
Effects of oroxylin A on LPS-induced expression of IL-1β and IL-6 in BV-2 cells. BV-2 cells (5×10^4^ cells/well) were co-treated with LPS (100 ng/ml) and oroxylin A (Oro-A, 50 µM). Effects of oroxylin A on LPS-induced increase of IL-1β and IL-6 mRNA were determined in (**A**) 1 hour and (**B**) 20 hours by real-time RT-PCR. Results are expressed as fold increase of target mRNA relative to the mRNA level in 0 hour, and are mean±SEM from three independent experiments. n.s., not significant, compared with LPS-treated cells. *, p<0.05, Student's t-test compared with LPS-treated cells.

The possibility that oroxylin A inhibited both the early and the late expression of IL-1β and IL-6 was examined. Incubation of BV-2 cells with LPS (100 ng/ml) for 1 hour resulted in 70- and 30-fold induction of IL-1β and IL-6 mRNA (the early expression), respectively (Fig. 4A). Both inductions were not significantly affected by its co-treatment with oroxylin A (50 µM). However, 20 hours after LPS treatment, the induction of IL-1β (300-fold) and IL-6 mRNA (1500-fold) by LPS (the late expression) was reduced significantly by co-treatment with oroxylin A (Fig. 4B).

### Oroxylin A did not affect LPS-induced nuclear accumulation of NFκB-p65

**Figure 5:**
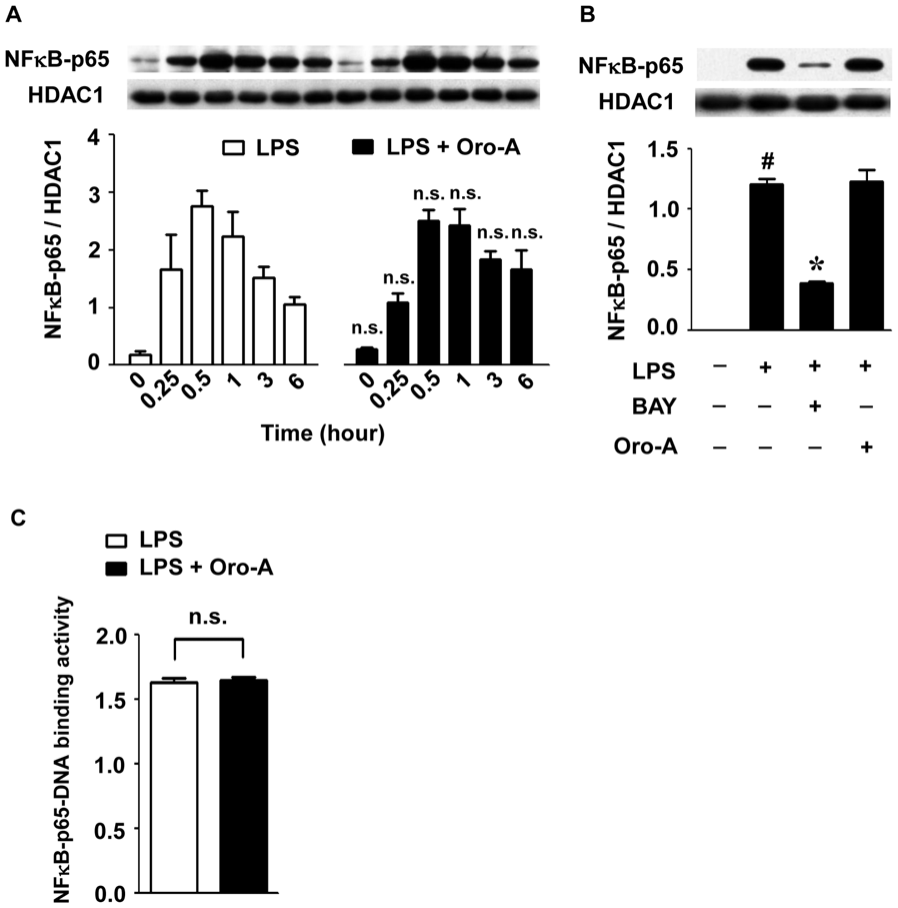
Failure of oroxylin A to inhibit LPS-induced activation of NFκB-p65 in BV-2 cells. (**A**) BV-2 cells (2×10^6^ cells/dish) were incubated with LPS (100 ng/ml) and oroxylin A (Oro-A, 50 µM) for indicated time periods. Nuclear proteins were then isolated and examined by Western blot analysis using specific antibodies against NFκB-p65 and HDAC1. HDAC1 is used as nuclear internal control here. A representative Western blot for NFκB-p65 and HDAC1 is shown in upper panel. The ratio of NFκB-p65 to HDAC1 is calculated and shown in bottom panel. (**B**) Cells (2×10^6^ cells/dish) were incubated with a NFκB inhibitor, BAY 11-7082 (BAY, 5 µM), or oroxylin A (Oro-A, 50 µM) in the presence of LPS (100 ng/ml) for 30 minutes. Nuclear proteins were then isolated and examined by Western blot analysis using specific antibodies against NFκB-p65 and HDAC1. A representative Western blot for NFκB-p65 and HDAC1 is shown in upper panel. The ratio of NFκB-p65 to HDAC1 is showed in bottom panel. (**C**) Cells (2×10^6^ cells/dish) were exposed to LPS (100 ng/ml) and oroxylin A (50 µM) for 30 minutes. Nuclear proteins were then isolated and subjected to transcription factor DNA-binding activity assay. All results are expressed as means±SEM from three independent experiments. n.s., not significant, compared with LPS-treated cells. #, p<0.05, one-way ANOVA followed by Tukey's post hoc test compared with vehicle-treated cells; *, p<0.05, one-way ANOVA followed by Tukey's post hoc test compared with LPS-treated cells.

After LPS (100 ng/ml) stimulation, NFκB-p65 proteins in the nucleus significantly increased in 15 minutes and reached the peak in 30 minutes (Fig. 5A). Thereafter, the level of nuclear NFκB-p65 declined while LPS was still present in the medium, although it was still higher than that of the control group in 6 hours after LPS treatment. This LPS-induced time-dependent nuclear accumulation of NFκB-p65 was not significantly affected by oroxylin A (50 µM) at any time point measured after LPS treatment. As a positive control, BAY 11-7082 (5 µM), a NFκB inhibitor [23], significantly attenuated the nuclear translocation of NFκB-p65 (Fig. 5B).

### Oroxylin A did not affect NFκB-p65 DNA-binding activity

We further determined whether the DNA-binding activity of NFκB-p65 in the nucleus was reduced by oroxylin A. NFκB-p65 DNA-binding activity was significantly increased 30 minutes after LPS stimulation as compared to that in 0 minute (data not shown). This increase of NFκB-p65 DNA-binding activity was not significantly affected by oroxylin A (50 µM, Fig. 5C).

### Oroxylin A inhibited LPS-induced activation of STAT1

**Figure 6:**
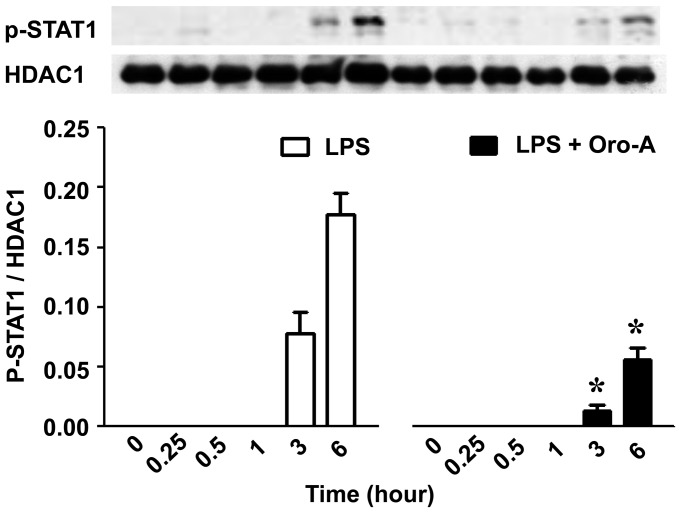
Oroxylin A inhibition of LPS-induced STAT1 activation in BV-2 cells. BV-2 cells (2×10^6^ cells/dish) were co-treated with LPS (100 ng/ml) and oroxylin A (Oro-A, 50 µM) for indicated time periods. Nuclear proteins were then isolated and subjected to Western blot analysis using specific antibodies against p-STAT1 and HDAC1. A representative Western blot is shown in upper panel and the ratio of p-STAT1 to HDAC1is calculated and shown in bottom panel. Results are expressed as means±SEM from three independent experiments. *, p<0.05, Student's t-test compared with corresponding LPS-treated cells.

**Figure 7:**
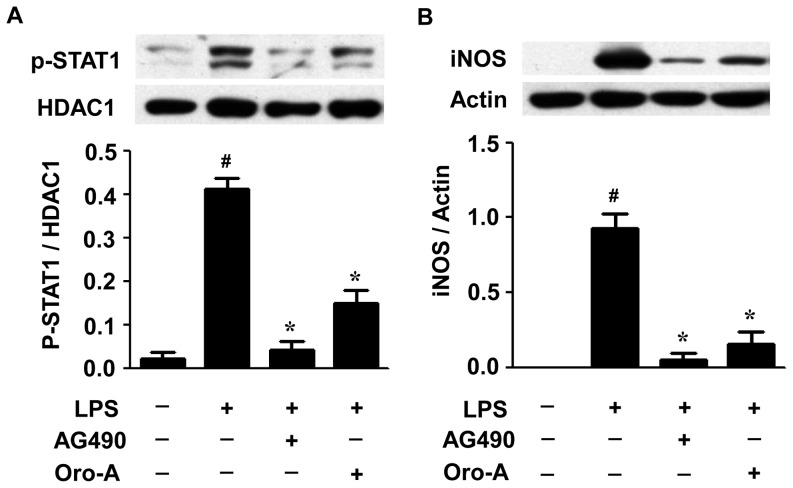
Effect of AG490 and oroxylin A on LPS-induced STAT1 phosphorylation and iNOS expression in BV-2 cells. (**A**) BV-2 cells (2×10^6^ cells/dish) were incubated with AG490 (20 µM), a specific JAK2 inhibitor, or oroxylin A (Oro-A, 50 µM) in the presence of LPS (100 ng/ml). After 16 hours, nuclear proteins were isolated and examined by Western blot analysis using specific antibodies against p-STAT1 and HDAC1. A representative Western blot is shown in upper panel. The ratio of p-STAT1 to HDAC1 is calculated and shown in bottom panel. (**B**) Cells (2×10^6^ cells/dish) were treated with LPS (100 ng/ml) and AG490 (20 µM)/oroxylin A (50 µM) for 16 hours. Effects of AG490 and oroxylin A on LPS-induced iNOS protein expression were determined by Western blot analysis. A representative Western blot is shown in upper panel and the ratios of iNOS to actin are shown in panel below. All results are expressed as means±SEM from three independent experiments. #, p<0.05, one-way ANOVA followed by Tukey's post hoc test compared with vehicle-treated cells; *, p<0.05, one-way ANOVA followed by Tukey's post hoc test compared with LPS-treated cells.

Different from the activation time course of NFκB-p65, the phosphorylation of STAT1 induced by LPS (100 ng/ml) was not observed until 3 hours after LPS (100 ng/ml) challenge, and the level of STAT1 phosphorylation was even higher 6 hours after LPS challenge (Fig. 6). Oroxylin A (50 µM) significantly reduced the STAT1 phosphorylation 3 and 6 hours after LPS challenge by 85% and 70%, respectively. Since phosphorylation of STAT1 is dependent on the kinase activity of JAK2 [18], effects of AG490, a specific JAK2 inhibitor [18], on STAT1 phosphorylation in BV-2 cells was examined. AG490 (20 µM) significantly inhibited LPS-induced STAT1 phosphorylation (Fig. 7A) with almost completely diminished iNOS expression in BV-2 cells (Fig. 7B).

## Discussion

Activation of microglia is an important process involved in repairing brain injuries. Over-activation of microglia, however, can be highly detrimental to neuronal cells, due to release of several pro-inflammatory factors such as NO, IL-1β and IL-6 which are potentially neurotoxic [6], [8]. Accordingly, inhibition of microglia activation and production of pro-inflammatory factors may be beneficial in reversing microglia-mediated neurodegeneration. The present study demonstrated for the first time that oroxylin A via suppressing STAT1 phosphorylation inhibited LPS-induced expression of iNOS (and production of NO), IL-1β and IL-6 in microglial cells.

Our present findings are consistent with previous reports that oroxylin A attenuates LPS-induced NO production in RAW264.7 macrophages and microglial cells [19], [20]. In addition, inhibition of NO production in microglia reduces the loss of co-cultured neuronal cells [10], [24]. These results favor the hypothesis that oroxylin A is beneficial in microglia-mediated NO-induced neurotoxicity. NO synthesis is mainly catalyzed by iNOS in activated microglia [25]. In the present study, both up-regulated protein and mRNA levels of iNOS induced by LPS in BV-2 cells were attenuated by oroxylin A, further indicating that oroxylin A reduced NO production via inhibiting iNOS expression in BV-2 cells. This is consistent with that reported by others in macrophages and microglial cells [26]–[28].

Modulation of mRNA stability, i.e. the degradation rate of mRNA, is an important post-transcriptional mechanism in regulating iNOS gene expression [29]. It has been reported that c-Jun NH_2_-terminal kinase inhibitor reduced iNOS expression via facilitating its mRNA degradation in macrophages [30]. However, no appreciable effect of oroxylin A on the half-life of iNOS mRNA was found in BV-2 cells, suggesting that oroxylin A did not affect the process of iNOS mRNA degradation. This is consistent with reports by others demonstrating that inhibition of iNOS expression did not result from facilitating its mRNA degradation but from suppressing its transcription in macrophages and microglial cells [26]–[28]. Thus, oroxylin A reduction of iNOS mRNA is most likely due to its gene regulation at the transcriptional level.

It has been demonstrated that NFκB-p65 is required for the transcription of iNOS [31]. In the present study, neither LPS-induced NFκB-p65 translocation nor NFκB-p65-DNA binding activity was affected by oroxylin A, suggesting that oroxylin A inhibition of LPS-induced iNOS expression is unlikely resulted from suppressing NFκB-p65 activation. This is consistent with reports by others showing that attenuation of LPS-induced iNOS expression is not necessarily accompanied by suppressing NFκB-p65 activation in macrophages or glial cells [17], [18], [28], [32].

It has been reported that LPS-induced IFNs mediate the phosphorylation of STAT1 which is involved in controlling the timing of iNOS expression [15]. It also has been shown that AG490, a specific JAK2 inhibitor, via inhibiting the phosphorylation of STAT1, attenuated LPS-induced iNOS expression [18]. These results suggest that JAK2-mediated STAT1 phosphorylation plays an important role in iNOS expression. In the present study, we also found that AG490 inhibited LPS-induced STAT1 phosphorylation resulting in attenuation of iNOS expression in BV-2 cells. Interestingly, LPS-induced STAT1 phosphorylation was inhibited by oroxylin A. Accordingly, oroxylin A attenuation of LPS-induced iNOS expression is likely via inhibiting STAT1 phosphorylation in BV-2 cells.

We, however, reported previously that oroxylin A attenuation of LPS-induced iNOS expression in RAW264.7 macrophages was mediated by inhibiting NFκB-p65 activation [19]. The exact reason for the difference between effects of oroxylin A on RAW264.7 macrophages and BV-2 cells is not known. It, however, has been reported that responses to the same stimulus of microglia and macrophages, which are derived from the same origin, can be distinct based on the differences in their gene expression and function [33]. It appears that oroxylin A acts in a cell-type-dependent manner. The cell-dependent phenomena for several other compounds have been reported. For instance, cAMP inhibition of LPS-induced iNOS expression in C6 glioma cells was via suppressing the phosphorylation of p38-MAPK, while in RAW264.7 macrophages LPS-induced p38-MAPK phosphorylation was not affected by cAMP which, on the contrary, up-regulated iNOS expression [34]. Moreover, 15-deoxy-delta 12,14-prostaglandin J_2_ (15d-PGJ_2_) inhibited LPS-induced iNOS expression without affecting NFκB-p65 translocation or NFκB-p65-DNA binding activity in microglial BV-2 cells [35]. In RAW264.7 macrophages, however, inhibition of iNOS expression by 15d-PGJ_2_ was associated with suppression of NFκB-p65 nuclear translocation [36].

Here, we showed that LPS induced the early expression of IL-1β and IL-6 in 1 hour and in 20 hours (the late expression) after LPS challenge in BV-2 cells. However, only the late expression of IL-1β and IL-6 was inhibited by oroxylin A. It is well known that expression of IL-1β and IL-6 is regulated by both NFκB and STAT1 [37]. The timing of early expression of IL-1β and IL-6 was correlated to that of NFκB-p65 activation (Fig. 5), suggesting that NFκB-p65 is involved in the early expression of IL-1β and IL-6. The finding that oroxylin A did not affect LPS-induced activation of NFκB-p65 provides an explanation that LPS-induced early expression of IL-1β and IL-6 was not inhibited by oroxylin A. On the other hand, activation of STAT1 began 3 hours after LPS stimulation, indicating that STAT1 is not likely associated with LPS-induced early expression of IL-1β and IL-6. However, it was demonstrated that JAK2 knockdown resulted in suppression of LPS-induced production of IL-1β and IL-6 by 24 hours [38], suggesting that JAK2-STAT1 signaling pathway is involved in late expression of these two pro-inflammatory cytokines. This is consistent with the present findings that oroxylin A inhibition of STAT1 activation was accompanied by suppressing LPS-induced expression of IL-1β and IL-6 20 hours after LPS challenge. It is likely that oroxylin A, via inhibiting STAT1 activation, suppresses the late expression of IL-1β and IL-6. In this regard, it is interesting to note that over-expressing suppressor of cytokine signaling 1 (SOCS1), which was demonstrated to inhibit STAT1 but not NFκB activation, inhibited LPS-induced late production of tumor necrosis factor (TNF) and IL-6, while the early production of TNF was not affected [39].

In conclusion, we demonstrated that oroxylin A inhibited LPS-induced activation of BV-2 miroglial cells. Oroxylin A attenuation of production of NO and expression of iNOS, IL-1β and IL-6 was via suppressing STAT1 phosphorylation. Since massive activation of microglia with excessive production of pro-inflammatory factors is associated with inflammation-induced neuronal death [8], our results suggest that oroxylin A may be beneficial in inflammation-induced microglia-mediated neurodegeneration.

## Materials and Methods

### Cell culture

The murine microglial cell line BV-2 originally developed by Dr. Blasi [40] was generously provided by Dr. Liang YC (School of Medical Laboratory Science and Biotechnology, Taipei Medical University, Taipei, Taiwan). BV-2 cells were cultured and maintained in Dulbecco's modified Eagle's medium (DMEM; HyClone, Logan, UT, USA) containing heat-inactivated 10% fetal bovine serum (FBS; Invitrogen, Carlsbad, CA, USA) and antibiotics (100 units/ml penicillin G and 100 units/ml streptomycin; Invitrogen) at 37°C in a humidified incubator under 5% CO_2_. Upon confluence, BV-2 cells were sub-cultured in a 24-well plate or 10-cm Petri dish for various experimental purposes. In all experiments, BV-2 cells were incubated in DMEM containing 2% FBS and treated with or without lipopolysccharide (LPS; Sigma-Aldrich, St. Louis, Missouri, USA) in the presence or absence of oroxylin A. In examining effects of oroxylin A on LPS-induced activation of BV-2 cells, oroxylin A and LPS were co-treated at the same time.

### Griess reaction

BV-2 cells cultured in 24-well plates at 5×10^4^ were incubated with oroxylin A (10–100 µM) and LPS (100 ng/ml) for 24 hours. The nitrite concentrations in the culture medium, indicative of NO production, were measured colorimetrically by Griess reaction [19]. Briefly, 100 µl of culture medium was mixed with an equal volume of Griess reagent (1% sulfanilamide/0.1% N-1-naphthyl)-ethyl-enediaminedihydrochloride/2.5% H_3_PO_4_; Sigma-Aldrich). After five-minute incubation, the absorbance at 550 nm was determined using a microplate reader (Molecular Devices, Sunnyvale, CA, USA). Sodium nitrite was used as a standard to calculate the concentration of nitrite in culture medium.

### MTT assay

The cell viability was measured colorimetrically using 3- (4,5-cimethylthiazol-2-yl)-2,5-diphenyl tetrazolium bromide (MTT, Sigma-Aldrich). MTT is actively catalyzed by mitochondrial succinate dehydrogenase to form formazan in live cells. Formation of formazan is therefore used as an indicator of the cell viability. BV-2 cells cultured in 24-well plates at 5×10^4^ were treated with oroxylin A and LPS for 24 hours, followed by incubation of MTT (0.5 mg/ml) for additional 4 hours. The formazan formed in cells were dissolved by dimethyl sulfoxide (DMSO; Sigma-Aldrich) and read at 565 nm using a microplate reader.

### Western blot analysis

BV-2 cells were cultured in 10-cm Petri dish at 2×10^6^ and treated with oroxylin A (10–100 µM) and LPS (100 ng/ml) for various time periods. At indicated time points, BV-2 cells were lysed using PRO-PREP™ protein extraction solution (iNtRON Biotechnology, Seoul, Korea) to collect total protein extracts, or using the Nuclear Extract Kit (Active Motif, Tokyo, Japan) to collect nuclear protein extracts according to the procedure described by the manufacturer. The protein concentration was measured using BCA protein assay kit (Pierce, Rockford, IL, USA). Equal amount of protein samples were separated on 10% SDS polyacrylamide gels. Proteins were then transferred onto a polyvinylidene difluoride (PVDF; Millipore) membranes using a ECL Semi-Dry Transfer Unit (Amersham Biosciences, Piscataway, NJ, USA) and subsequently blocked for 2 hours at room temperature with 5% non-fat milk in Tri-buffered saline containing 0.25% Tween (TBST). Membranes were then incubated overnight at 4°C with specific antibodies for iNOS (1∶1000; Chemicon, Temecula, CA, USA; Cat. # 610432), NFκB p65 (1∶500; Santa Cruz, CA, USA; Cat. # sc-8008), phospho-STAT1 (Tyr701) (1∶1000; Cell Signaling Technology Beverly, MA, USA; Cat. # 9171), HDAC1 (1∶2000; Biovision, Mountain View, CA, USA; Cat. # 3601) or actin (1∶10000; Millipore, Billerica, MA, USA; Cat. # MAB1501) in 5% non-fat milk. After washing with TBST, membranes were incubated with horseradish peroxidase (HRP)-conjugated anti-mouse or anti-rabbit secondary antibodies (1∶2000; KPL, Gaithersburg, MD, USA) for 2 hours at room temperature and immumoreactivities were subsequently visualized using an enhanced chemiluminescence (ECL) detection method.

### Real-time reverse transcription-polymerase chain reaction (real-time RT-PCR)

BV-2 cells cultured in 24-well plates were incubated with oroxylin A (50 µM) and LPS (100 ng/ml) for indicated time periods. Total mRNA was isolated from BV-2 cells using TRI reagent (Applied Biosystems, Foster City, CA, USA) according to manufacturer's instructions. The amount of mRNA was quantified using spectrophotometer, and 1 µg of mRNA were reversely transcribed into first-strand cDNA by SuperScript™ III Reverse Transcriptase (Invitrogen) in a total reaction volume of 20 µl. Real-time PCR amplifications were performed in triplicate using mixture of 2× FastStar Universal SYBR Green Master (Roche Applied Science, Mannheim, Germany), 2 µl of cDNA samples and designate primers. The primers used were as follows: iNOS, sense: 5′-ACATCGACCCGTCAC- AGTAT-3′, antisense: 5′-CAGAGGGGTAGGCTTGTCTC-3′; IL-1β, sense: 5′-GAAATGCCACCTTTTGACAGTG-3′, antisense: 5′-CTGGA- TGCTCTCATCAGGACA-3′; IL-6, sense: 5′-TAGTCCTTCCTACCC- CAATTTCC-3′, antisense: 5′-TTGGTCCTTAGCCACTCCTTC-3′; actin, sense: 5′-GGCTGTATTCCCCTCCATCG-3′, antisense: 5′-CCAGTTG- GTAACAATGCCATGT-3′. The real-time PCR were performed for 45 cycles of 95°C for 15 s and 60 for 1 minute using a ABI Prism 7300 instrument (Applied Biosystems).

### iNOS mRNA stability assa*y*

BV-2 cells culture in 24-well plates were stimulated with LPS (100 ng/ml) for 20 hours, and then treated with a transcription inhibitor actinomycin D (0.1 µg/ml; Tocris, Ellisville, MO, USA) in the presence of oroxylin A (50 µM) or vehicle for various time periods. Total mRNA from BV-2 cells was isolated at indicated time points and iNOS mRNA levels were quantified by Real-time RT-PCR. iNOS mRNA decay against time in the presence or absence of oroxylin A was then analyzed.

### Transcription factor DNA-binding activity assay

DNA-binding activity of NFκB-p65 was analyzed using NFκB (p65) Transcription Factor Assay Kit (Cayman Chemical, Ann Arbor, MI, USA) according to manufacturer's instructions. Briefly, nuclear proteins were extracted from BV-2 cells after LPS (100 ng/ml) and oroxylin A (50 µM) treatments for various time periods. Equal amounts of nuclear extracts were incubated overnight in a 96-well plate coated with a NFκB consensus double-stranded DNA (dsDNA). A competitor dsDNA was added to confirm the DNA-binding specificity of NFκB. The samples were then incubated with primary NFκB-p65 antibody for 1 hour. Subsequently, they were incubated with HRP-conjugated secondary antibodies for an additional hour. After the incubation of developing solution, the reaction was stopped by a stop solution, and the absorbance at 450 nm was measured by microplate reader.

### Statistical analysis

All experiments were performed at least 3 times and the data were expressed as means±SEM. Statistical significance was analyzed by one-way ANOVA followed by Tukey's post hoc test or Student's t-test. A value of P<0.05 is considered statistically significant.
